# The Role of Adipokines in Pancreatic Cancer

**DOI:** 10.3389/fonc.2022.926230

**Published:** 2022-07-08

**Authors:** Qi Wang, Huizhi Wang, Yuntao Ding, Mengtian Wan, Min Xu

**Affiliations:** Department of Gastroenterology, Affiliated Hospital of Jiangsu University, Jiangsu University, Zhenjiang, China

**Keywords:** obesity, pancreatic cancer, adipokines, white adipose tissue, leptin, adiponectin, chemerin

## Abstract

In modern society, inappropriate diets and other lifestyle habits have made obesity an increasingly prominent health problem. Pancreatic cancer (PC), a kind of highly aggressive malignant tumor, is known as a silent assassin and is the seventh leading cause of cancer death worldwide, pushing modern medicine beyond help. Adipokines are coming into notice because of the role of the intermediate regulatory junctions between obesity and malignancy. This review summarizes the current evidence for the relationship between highly concerning adipokines and the pathogenesis of PC. Not only are classical adipokines such as leptin and adiponectin included, but they also cover the recognized chemerin and osteopontin. Through a summary of the biological functions of these adipokines as well as their receptors, it was discovered that in addition to their basic function of stimulating the biological activity of tumors, more studies confirm that adipokines intervene in the progression of PC from the viewpoint of tumor metabolism, immune escape, and reprogramming of the tumor microenvironment (TME). Besides endocrine function, the impact of white adipose tissue (WAT)-induced chronic inflammation on PC is briefly discussed. Furthermore, the potential implication of the acknowledged endocrine behavior of brown adipose tissue (BAT) in relation to carcinogenesis is also explored. No matter the broad spectrum of obesity and the poor prognosis of PC, supplemental research is needed to unravel the detailed network of adipokines associated with PC. Exploiting profound therapeutic strategies that target adipokines and their receptors may go some way to improving the current worrying prognosis of PC patients.

## Introduction

The poor prognosis among subsistent solid tumors due to their atypical early clinical symptoms, high local aggressiveness, distant metastasis by the time of clinical diagnosis, and the absence of effective treatment has rendered pancreatic cancer (PC), a kind of highly aggressive malignant tumor, known as a silent assassin and the seventh leading cause of cancer death worldwide (1, 2). By 2025, scientific models predicted that PC would surpass breast cancer as the third reason accounting for the mortality of tumors (3). The prevalence of PC keeps increasing, supported by comparative analysis of data from 48 countries, and this gain in prevalence is obvious both in the population aged 50 years or older and in younger age groups (4). While medical advances have led to effective control of the mortality of multiple cancer groups in both sexes, the latest European cancer death rate projections also suggest that the patient population of PC presents the opposite trend where mortality and prognosis are unpromising and PC is the next central public health problem facing Europe and the world (5). A brief conclusion suggesting that suboptimal populations with smoking, physical inactivity, alcohol consumption, and obesity account for a large proportion of the PC population could be summarized from a series of epidemiological studies, and the occurrence of PC reflects, in part, the increasing prevalence of obesity, diabetes, and alcohol consumption (2, 6, 7).

The obese population, building on this foundation, makes up a large part, with obese men accounting for roughly 6% of PC cases and obese women with a proportion of 7% (8). Among the 13 malignancies for which the risk of oncogenesis in humans is causally related to physical obesity is pancreatic cancer (9, 10). Body mass index (BMI), an estimation of the degree of obesity, which increases the risk of PC by 10% for every 5 of a unit increase, appears to indicate a greater vulnerability to PC in later life when an individual has a high BMI in adulthood (8). Obesity is deemed as a considerable causative factor for PC at all time periods in the human body (11, 12). As a global epidemic, obesity is accompanied by susceptibility to various maladies (13). Preventing obesity is as crucial as controlling tobacco intake in controlling the morbidity and mortality of pancreatic cancer and improving the well-being of patients.

The level of body fat in obese groups often reaches dysfunctional levels where it may alter the physiological regulation of adipose tissue (AT), leading to obesity-related disorders that include cancer. It was believed that AT, as an inert organismal storage tissue, was mainly involved in the energy supply of the body and in the regulation of body temperature and other basic physiological activities (14). With a deeper understanding of the excessive fat deposition arising from obesity and the pathological expansion of white adipose tissue (WAT), there exists a consensus that the chronic low inflammatory state caused by AT has a powerful link with the recruitment of immune/inflammatory cells, the induction of neovascularization, the regulation of tumor-associated adipose tissue microenvironment (TAAME), as well as cancer-associated adipocytes (CAAs), and other processes contributing to cancer development (15, 16). AT is no longer simply a receptacle for fat storage, but has emerged as an endocrine organ capable of producing various hormones and factors (17, 18). The concept of the adipokine family has evolved from the earliest known leptin and adiponectin to the recognized omentin and chemerin, which are the main molecular links between AT and the regulation of metabolic syndromes, cardiovascular diseases, and various malignancies, and are integrated with other cytokines to form a sophisticated functional network (19). Relying on novel proteomics techniques or methods, this family is expanding and more novel adipokines are being discovered, leading many scholars to pay more attention to the relationship between adipokines and tumors (20).

Given the unique mediating functions of adipokines in the management of physiological responses in AT, the regulation of their secretion levels or the modulation of their corresponding receptors may be a key element in the progression of PC (**Table 1****)**. An in-depth investigation of the mechanisms of adipokines in relation to carcinogenesis may become an invaluable field for the earlier clinical detection of PC and the advancement of the prognosis of PC patients. This review summarizes the latest insights into the dysfunction of the body brought about by the structural and biological properties of AT and the adipokines it produces. It also presents a review of the classical and novel adipokines identified to act in the pathogenesis of PC. Furthermore, this paper explores the subtle interactions between the adipose tissue microenvironment and the tumor microenvironment (TME), thus reflecting on possible orientations for future studies on adipokines in tumor research, which is vital for proposing innovative ideas in clinical treatment.

**Table 1:** Effect of Adipokines on PC.

Adipokines	Receptor	Expression	Link	Effect	Signaling	References
Leptin	LEPR	↑	↑	Proliferation (+) Migration (+) Invasion (+) Durg resistance (+)	JAK2/STAT3, PI3K/AKT Notch	(21, 22) (23–25)
Adiponectin	AdipoR1 AdipoR2	↓ (large-scale) ↑ (small-scale)	↓	Proliferation (−) Migration (–) Invasion (–) Pro-apoptotic (+)	Caspase β-Catenin	(26–31)
Resistin	TLR4 CAP1	↑	↑	Proliferation (+) Migration (+) Invasion (+) Durg resistance (+) Pro-inflammatory (+)	STAT3	(32)
Oncostatin	gp130/LIFRβ gp130/OSMRβ	↑	↑	Proliferation (+) Migration (+) Invasion (+) Durg resistance (+) Pro-inflammatory (+) TME reprogramming (+)	JAK/STAT3 ERK2/Sp1	(33–37)
Osteopontin	OPN- integrin- CD44	↑	↑	Proliferation (+) Migration (+) Invasion (+) TME reprogramming (+)Immune escape (+)	Akt/Erk FOXM1/ H3K4me3	(38–41) (42–45)
Kisspeptin	KISS-1R (Gpr54)	↓ (Tissue) ↑ (Serum)	↑	Proliferation (–) Migration (+) Invasion (+)	ERK1/p38	(46–50)
Omentin	Unknown	↑	↑	Unknown	Unknown	(51, 52) (53, 54)
Chemerin	Chemerin 1 Chemerin 2 CCRL2	↑	↑	Unknown	Unknown	(53)

**ANNOTATION : **↑ mean promote, **↓** mean suppress; The **link** column refers to the impact on PC development; **(+)** mean exist (–), mean absent; The **Effect** column refers to the specific outcome of adipokines to PC.

## Adipose tissue

AT, perhaps preferably called adipose organs, seems to be a dynamic tissue complex composed of adipocytes and interstitial vascular components, which both form an intangible portion of the human body and comprise the TME. AT is mainly composed of WAT, brown adipose tissue (BAT), and beige adipose tissue (55, 56). Of these, WAT produces cytokines and chemokines that underlie its biological function, while BAT, which is in a hyper-metabolic state and capable of heat production, relies on a specific metabolic pathway that is thought to be associated with the activation of UCP1 (57).

### BAT and batokines

The distinction lies within the three categories of AT is the content of cellular mitochondria and the higher the relative quantity of mitochondria AT possesses, the darker color it tends to exhibit (58). BAT, which contains multilocular lipid droplets as well as a substantial portion of mitochondria and expresses mitochondrial UCP1, turns out to be the central heat generating plant of the human body (59). Beige adipose tissue is phenotypically similar to BAT and is transformed from WAT after exposure to cold stimulation or β3-adrenergic agonists (60, 61). The distribution of beige adipose tissue in the body differs according to the stimulation of the location of WAT and is highly heterogeneous (62).

Despite the thermogenic properties of both beige adipose tissue and BAT, which serve an instrumental function in the adjustment of systemic energy homeostasis, beige adipose tissue and BAT, drawing inspiration from the endocrine function of WAT, may also be participating in the management of human ailments through the release of batokines (62). Batokines represent a collection of active peptides generated by BAT under its natural or thermogenic activation state, and it is shown that BAT is engaged in the regulation of systemic metabolism and cardiac, hepatic, and pancreatic function *via* releasing batokines, such as FGF21, BMP8b, NRG4, IL-6, and IGF-1 (63, 64). FGF21, being the earliest discovered batokine, has acquired attention for the delayed ventricular remodeling effect in hypertensive heart disease (65). Nevertheless, FGF21 is equally protective of the pancreas, as evidenced by its capacity to halt adipose deposition in the pancreas and chronic inflammation of the pancreas, thereby effectively limiting the malignant progression of PC (66). Alongside assisting BAT in thermogenesis, batokines could reduce the local inflammatory response by restraining immune cell activities, building upon the established practice that the thermogenic function of BAT is antagonistic to its pro-inflammatory properties (67). IL-6 is known to stand out as a key contributor to inflammation, oncology, and various metabolic disorders (68, 69). CXCL14 is released by BAT upon its thermogenic activation and ample proof has demonstrated its recruitment to M2 macrophages in diverse types of AT (70). GDF15, another batokine, performs the final loop of anti-inflammatory properties (67, 71). Relying on these above elements, which have been well documented in the association of tumors in previous studies, BAT communicates with the function of the liver, heart, and immune system frequently (63). However, a limited amount of research remains on the linkage between BAT and beige adipose tissue and PC, meaning that further studies are required to explore the underlying mechanisms involved and unravel the myth of metabolic health enhancement capabilities. The three types of AT could be converted into each other, which is referred to as the plasticity of AT (72). Beige adipose tissue is known to be derived from WAT, whereas BAT also transforms into white/beige adipose tissue when faced with elevated ambient temperature, the deficiency of leptin receptor, the impairment of β-adrenergic signaling, and the lack of lipase (73).

### White Adipose Tissue

#### WAT Related Endocrine Function

Regrettably, in spite of the widespread recognition of the participation of obesity and AT in the occurrence and development of PC, the understanding of the mechanistic principles involved remains at a relatively low level. WAT serves as a major agent of metabolism and inflammation, simultaneously modulating metabolism by balancing energy expenditure, adipocyte differentiation, and insulin sensitivity, along with the production of pro-inflammatory and anti-inflammatory molecules and the activation of immune signaling to regulate inflammation (74). In addition to the meta-inflammation attributed to WAT, the production and secretion of adipokines account for the core function of the WAT, and furthermore, the relationship between its endocrine properties and meta-inflammation is well established. The identification of leptin unlocked the floodgates of recognition of adipokines as WAT secretagogues, and adipokines were subsequently proved to be a sequence of heterogeneous polypeptides that participate in the regulation of lipid metabolism, human immunity, insulin resistance, inflammation reaction, angiogenesis, carcinogenesis, and other pathophysiological processes (75–77). And as the fields of metabolomics, proteomics, and genomics are being explored, over 100 members are constantly being added to this family. The network of adipokines relevant to neoplastic progression is being expanded, and the list currently contains 15 adipokines (20). Distinct adipokines bound to their respective receptors are distributed in a wide range of organs and tissues, interfacing and regulating diverse signaling pathways in neoplastic cells (78). Adipokines exhibit context-dependent interactions with diverse cancers, so it is not surprising, for instance, that even though adiponectin is proven to have a guardian angel effect in an otherwise malignant tumor, including breast cancer, the same does not hold true in clinical studies or mechanistic studies concerning PC (79, 80). Alongside with the increasing acceptance of the idea of adipokines as therapeutic targets, more in-depth research is being conducted For instance, immunotherapy may contribute to the increased immune response stimulated by immunotherapy in cancer patients. There are implications of adipokines for reprogramming of TME (78, 81).

#### WAT in Various Depots

Via distinct signing mechanisms, AT relates to additional systemic components through its secreted substances. The impact of AT at either the systemic or local scale is well explained when considering it as a collection of depots scattered throughout the body, with separate depots comprising the whole being individually focused on various features, even more precipitated diverse gradients of disease risk (82, 83). The subcutaneous adipose tissue (SAT), located underneath the skin, and the visceral adipose tissue (VAT), surrounding the internal organs of the body, together constitute the bulk of WAT. Both deposits have separate metabolic specificity (51). VAT containing substantially more immune cells displays increasingly active metabolic traits, accompanied by elevated insulin resistance and expression of inflammatory mediators or adipokines, which is considered convincing evidence for engaging VAT in obesity-related metabolic disorders or diseases, such as cancer (51, 84). One compelling argument shows that VAT levels inversely correlate with serum adiponectin, of which the anti-inflammatory and anti-cancer functions have garnered enough attention, as shown by the mechanisms described below (85). The direct impact exerted by VAT on the endocrine system could consequently induce metabolic disorders. This, moreover, could partly mirror the assessment of disease prognosis. Not only is VAT measurement an invaluable element in the assessment system for estimating the effectiveness of chemotherapy in PC patients, but it is equally applicable to patients after PC surgery, both signifying alarming prognostic consequences (86, 87).

Given that SAT and VAT, described above, appear to be indirectly and teleologically regulated in the case of PC, scholars are increasingly focusing on intrapancreatic fat within the setting of PC, a pancreas-specific fat storage reservoir (19). Therefore, AT may prefer to interact in a somewhat direct and paracrine mode to promote carcinogenesis. AT-related inflammation and metabolic disorders of paracrine secretion are followed by the over-fat deposit in the pancreas, which exceeds a certain common threshold (88). The pathological accumulation of AT in the pancreas or replacement of pancreatic alveolar cells by adipocytes following unnatural death both facilitate the progression of fatty pancreas (89). Intrapancreatic adipose concentrations have recently been recognized as a risk factor for early PC lesions and could shape the macroscopic or even microscopic environment of PC, with the potential of being a key criterion for early cancer detection (90, 91). Infiltration of adipocytes and an increase in adiposity are detectable in PC tissue, which suggests a poor prognosis (88, 92).

The distinct localization of VAT or intrapancreatic fat might determine how its secreted adipokines act on PC, which may involve an endocrine pattern or a paracrine manner to generate local tissue inflammation or, more likely, a combination of these two heterotypic signaling (89, 93). Adipocytes constitute a central stromal component of the tumor microenvironment with the ability to interact with cancer cells through cellular autonomous signaling together with paracrine messaging by secreting multiple regulatory molecules, notably adipokines (94). Such signaling mechanisms are seen to be involved in KRAS-mediated reprogramming of TME metabolism, which in turn subtly alters the proliferative efficacy of pancreatic cells (95). OSM in TME is mainly secreted by tumor-associated macrophages (TAM) and AT with the capacity to induce VEGF production and angiogenesis, together with being a potent chemotactic agent for neutrophils, which consequently promotes inflammation-associated tumor progression (96). The pro-inflammatory properties of adipokines derived from the AT paracrine routes could contribute bidirectionally to the fibrotic and inflammatory condition of the pancreas and be a prospective contributor to the chemoresistance of PC (15). Another rather intuitive and intricate mechanism involves the secretion of adipokines by distantly located AT away from the tumor, which undergoes turnover in circulation and flow to reach the target organ, like the pancreas, for effect (94). Straightforward proof of this model is that existing sufficient studies to show adipokines such as leptin, resistin, and omentin are increased or even varied in serum of PC patients (21, 32, 52). For example, by interacting with its receptors, leptin acts as an endocrine agent *via* activating key molecules such as JAK2, STAT3, and PI3K and engaging in a wide range of signaling pathways, including JAK2/STAT3, PI3K/AKT, and Notch (97). The complex side of the endocrine approach, however, resides in the fact that at the macroscopic level of the human body, the over-secreted adipokines by AT carry with them metabolic disturbances, such as insulin resistance or glucose metabolism disturbances, which likewise impinge on PC (98, 99). Certainly, both endocrine and paracrine forms of adipokine action could operate on PC, more likely as a synergistic effect.

#### WAT Related Chronic Low-Grade Inflammation

Complicated chronic low-grade inflammation in WAT is triggered and maintained as a result of a dysfunctional secretion of inflammatory factors, cytokines, and chemokines, which is dependent on an immune response with a range of immune cells, and such ongoing inflammatory responses are thought to underlie tumorigenesis or other illnesses (100–102). Despite the recent suggestion that variation in mitochondrial function underpins the inflammatory effects of adipose tissue, one cannot ignore the contribution of immune cells to this pattern of inflammation (103). Macrophages are believed to be the primary regulators of WAT-associated chronic inflammation *via* playing major roles, including initiating the recruitment of additional immune cells and secreting cytokines to adjust the inflammatory signaling cascade in host organisms (74, 104). Stress-related involvement of NK cells and secreted cytokines engage closely in the inflammatory polarization of macrophages in AT (105). Glycolysis is seen as a major contributor to the pro-inflammatory properties of macrophages in AT, activating the release of adipokines from macrophages (106). CD11c (+) ATM (AT macrophages) in WAT induces the accumulation of macrophages in the liver and the subsequent persistence of inflammation, which is facilitated by the recruitment of CXCL14-expressing neutrophils (107). The translocation of monocytes to AT by monocytes themselves allows the maintenance of obesity-specific chronic inflammation to be possible (108). Neutrophils have also been shown to maintain the inflammatory process by the production of large amounts of IL-1β and induction of macrophage clustering and infiltration (109). An increase in AT T cells was clearly observed after high fat intake, and the increased T cells were characterized by high expression of IFN-γ, highly expressed immune mediators in AT, and IL-17 (74, 110). While the effects of T cells and their diverse isoforms on WAT-related chronic inflammation vary, intriguingly, it has been verified that obese individuals, often accompanied by insulin resistance, have remarkably reduced natural Tregs, which means that Tregs restrict the proinflammatory environment and hence reduce chronic inflammation in adipose tissue (74, 104, 111, 112). CD8(+) T cells are recognized to precede macrophage activation and to provoke alteration in their phenotype in order to launch the inflammatory response inflammation (74, 113). CD8(+) T cells have the capacity to amplify the scope of the inflammation following an inflammatory response. Kiran et al. (113) discovered an increase in the proliferation of activated CD8(+) and CD69(+) T cells following WAT expansion, together with enhanced CXCR3 receptor expression, alongside the observed recruitment and increased numbers of pro-inflammatory M1 macrophages, which maintained a low degree of chronic inflammation. Notably, this process probably also produces adipokines such as osteopontin and resistin, which are in tandem with the endocrine capabilities of WAT itself, as discussed further below (114, 115).

Such chronic low-grade inflammation complicated with WAT has the potential to form a closed loop above the mechanism that leads to an inflammatory cycle, maintaining or amplifying the inflammation of AT. Chronic inflammation with a high level of CRP or TNF-α receptor 2 prove to initiate the original of PC and make progression to the metastasis, pre-diagnostic proficiency of inflammatory biomarkers provably correlates negatively with pancreatic cancer survival (116). This meta-inflammation caused by obesity or WAT has been accepted to be related to the pancreas and its inflammation. Existing experimental testimony shows highly penetrant PC with a mutant KRAS gene, a critical oncogene in the initiation, proliferation and metabolic reprogramming of PDAC, develops fast when faced with food-induced obesity and inflammation (117). Chronic inflammation of the pancreas as a consequence of obesity is consistently regarded as a life-long hazard factor for increasing the morbidity of PC (118). The necessity in transformation of gene and the susceptibility to tumorigenesis of the mutation of KRAS brought with could be enhanced when hit by HFD and WAT-inflammatory, which will make PC more invasive (117). Gupta et al. (119) newly noted Endothelin-1 (ET-1), which is correlated with PC, can be upregulated in pancreatic tissue when come across the activation of KRAS, an oncogene that shows great effect in obesity, no matter chronically or acutely inflamed pancreas.

## Adipokines and pancreatic cancer

Previous studies in other cancer contexts, like BC and CRC, placed leptin, resistin, adiponectin, oncostatin, and osteopontin in the spotlight. Despite the acknowledged interactions of these five adipokines with PC, there remains no review to systematically cluster and analyze their contributions to PC. While leptin boosts the progression of PC through diverse signaling pathways, adiponectin could exert a protective effect that is rarely seen in other adipokines. Resistin serves as an essential biomarker in the pathophysiological progression of PC and is highly correlated with diagnosis and prognosis. However, research on oncostatin and osteopontin is more focused on the mechanisms of TME regulatory manipulation.

### Leptin

**Figure 1:**
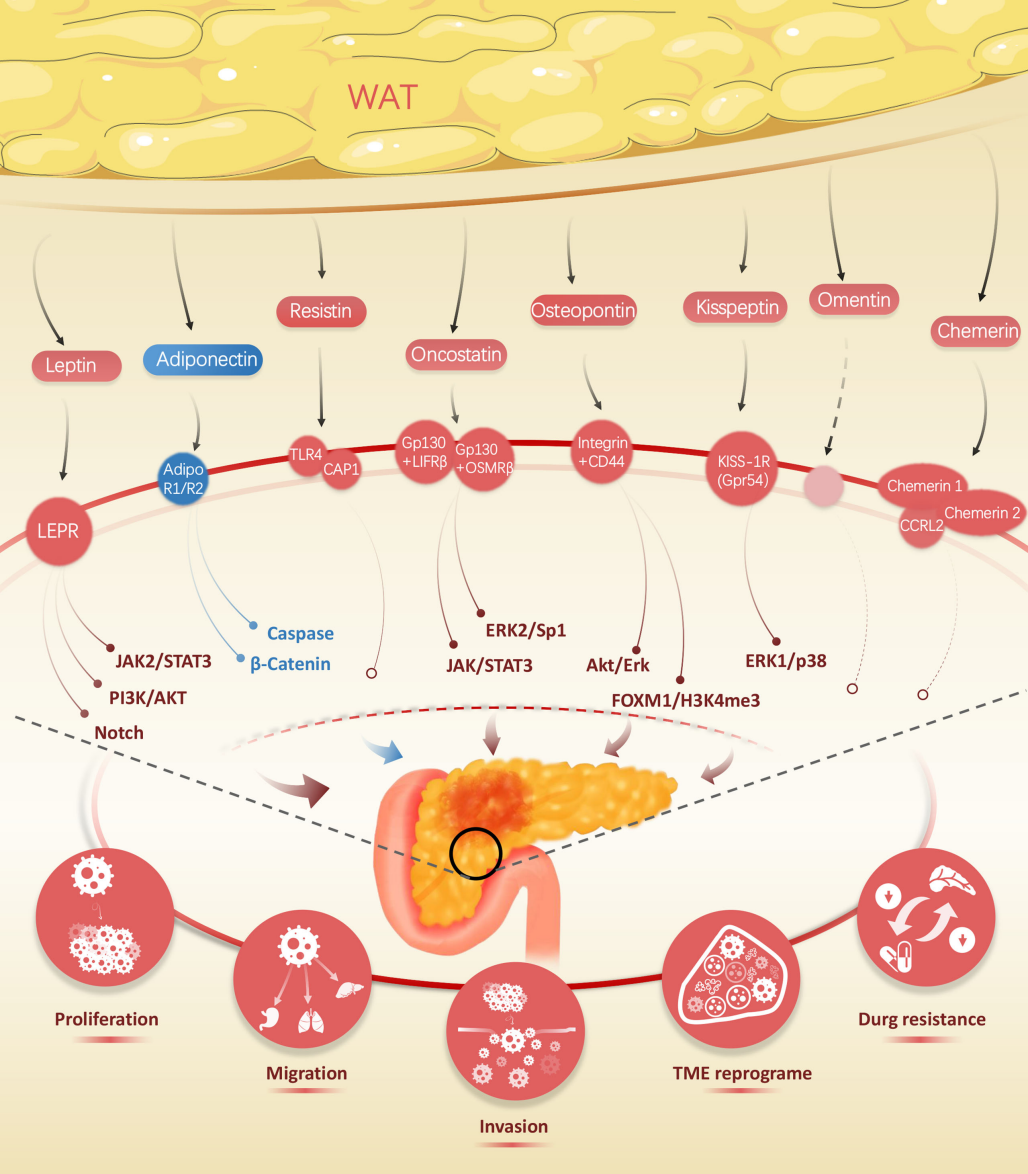
Wide range of adipokines released by white adipose tissue, including leptin, adiponectin, resistin, oncostatin, kisspeptin, omentin, and chemerin, impact on the biological traits of pancreatic cancer.

Leptin was initially discovered in 1994 by Zhang et al. (120) using a mouse cloning technique. Subsequently it was discovered that the protein was encoded by the obesity-related gene (OB Gene) and secreted by WAT (**Figure 1**). Following the advanced research on the mechanism of action of leptin, its functions were no longer constrained to the control of appetite and governance of energy balance by acting as a regulator of fat metabolism in the body, but more often as an adipokine to perform immune regulation, angiogenesis and intervention in the governance of tumor cell growth. Recently, it was shown that the modulation of tumor cell growth by leptin is related to the genesis and development of various tumors, among which PC is a current clinical hotspot given its poor prognosis and difficulty in diagnosis. White et al. (22) observed the relationship between leptin levels and PC growth in mice by controlling their body weight, suggesting that increased body weight significantly accelerated the growth of PC in mice and that the consequent increase in leptin levels was a potential mechanism by which obesity could affect the growth of PC. Stolzenberg-Solomon et al. (21) identified a significantly increased risk of occurrence of PC in patients followed for more than 10 years (OR = 2.55,95% CI = 1.23, 5.27; P = 0.004) by following up 731 cases of pancreatic adenocarcinoma that occurred between 1986 and 2010, which confirmed that leptin increased concentrations correlated with pancreatic carcinogenesis.

The mechanism of action of leptin occurs mainly through interaction with its specific receptors. Leptin receptor (LEPR, also known as ObRs), is a single transmembrane glycoprotein encoded by the LEPR gene and consists of six different isoforms of LEPR (a–f), which are widely distributed in the choroid plexus, hypothalamus, cardiovascular, liver, kidney, pancreas, and other organs throughout the body (121). Combination of LEPR with leptin invokes cancer progression by inducing molecular biological behaviors in normal tissue cells through EMT, cell–extracellular matrix interactions and extracellular matrix protein hydrolysis, which interrelate closely with tumor cell genesis, proliferation, distant migration, and apoptosis (122). Fan et al. (123) identified that leptin upregulates MMP-13 by mediating the JAK2/STAT3 signaling pathway, while the MMP family was previously proven to degrade various components of the extracellular matrix (e.g., basement membrane and fibronectin) in mutated cancers (ovarian, breast, etc.) with consequent promotion of tumor metastasis. In addition, it has been shown that MMP-13 positively relates to LEPR expression levels and that increased expression correlates substantially with lymph node metastasis in pancreatic cancer patients (123). Mendonsa et al. (124) identified LEPR short and LEPR long isoforms from PC tissues as well as confirmed that leptin specifically binds LEPR and activates the PI3K/AKT pathway to promote PC cell migration. Furthermore, the application of the AKT signaling pathway inhibitor LY294002 to PC cells significantly counteracted the effect of leptin on the proliferation of tumor cells, while the corresponding application of the AKT pathway activator IGF-1 counteracted the effect of leptin silencing, which similarly supports the notion that leptin can promote the migration and growth of PC by activating the PI3K/AKT signaling pathway (23). Notch receptors, ligands, and Notch-targeting molecules (Notch1-4, DLL4, JAG1, etc.) were highly expressed in PC cells cultured in leptin, while Notch expression levels were significantly reduced after administration of Notch inhibitory factor (DAPT) and leptin signaling inhibitor (IONP-LPrA2), indicating that Notch is required for leptin-induced PC proliferation (24). Also, the antigen expression of PC in the treated group was detected and remarkable expression of PCSC markers CD24/CD44/ESA, ALDH, CD133, and Oct-4 were found, suggesting that the leptin/Notch axis also affects PC stem cell progression (24). Notably, in a follow-up study, Harbuzariu et al. (25) found that leptin attenuated the cytotoxic effects of 5-fluorouracil (5-FU) on PC by increasing the expression of Notch signaling pathway transcription factors and eliciting increased expression of the drug efflux proteins ABCC5 and ABCC11, of which 5-FU retards chemoresistance precisely by decreasing ABCC5 and ABCC11, suggesting that the therapeutic sensitivity of 5-FU in PC patients could be strengthened clinically by targeting the leptin/Notch axis **(** **Table 1****)**.

### Adiponectin

Since its identification in 1995, adiponectin has attracted considerable attention due to its unique cancer-suppressive effects on common tumors such as colorectal, ovarian, and cervical cancers (125). Adiponectin is another endogenous bioactive product secreted by WAT and resides in the body chiefly in the form of low molecular trimers (LMW), medium molecular hexamers (MMW), and high molecular multimers (HMW), where the HMW exhibits the most intense activity as the main form of adiponectin activity (126). In the circulating blood, adiponectin exerts its activating effects on insulin sensitization, prevention of cardiovascular disease, diet regulation, and mediation of signal transduction pathways through binding with adiponectin receptors, including AdipoR1 (adiponectin receptor-1), expressed highly in skeletal muscle and liver, AdipoR2 (adiponectin receptor-2), localized to the liver, and T-cadherin, which is abundantly expressed in endothelial cells and smooth muscle cells (127) (**Figure 1**). Due to the specificity of the distribution of adiponectin receptors, current studies suggest that T-cadherin is participatory in atherogenesis, whereas AdipoR1 and AdipoR2 enriched in the liver, mainly moderating the role of signal transduction, are closely associated with procedures regarding insulin secretion and tumor metabolic reprogramming (128, 129).

Although adiponectin inhibits tumor progression by suppressing cell proliferation or inducing apoptosis in carcinomas of the cervix and breast, there is still much controversy regarding the relevance of adiponectin to PC and the underlying mechanism by which it impacts the PC. According to Dalamaga et al. (26), 81 cases of PC and 81 control healthy patients were included between 2000 and 2007 in the follow-up and it was concluded that high levels of adiponectin in the body were positively associated with the incidence of PC. Similarly, another clinical study, which measured serum adiponectin concentrations in 72 patients with PC, 39 patients with chronic pancreatitis, and 290 control patients, determined that median adiponectin levels were statistically higher in the PC group than in the control group (27). Both the above studies had relatively small sample sizes, yielding discrepancies in results after large sample studies. After 6 years of follow-up of 468 patients with PC and 1,080 healthy patients, Bao et al. (28) found that the median plasma adiponectin level of 6.2 µg/ml was lower in the PC group than in the control group at 6.8 µg/ml, implying a possible negative association between plasma adiponectin and PC risk. Based on the European Prospective Investigation into Cancer and Nutrition (EPIC cohort), Grote et al. (29) conducted a case–control study involving 452 PC patients and 452 healthy cases and confirmed that elevated adiponectin levels in never-smokers were negatively associated with PC risk (**Table 1****)**.

According to large-scale clinical studies, low concentrations of plasma adiponectin may be a risk factor for the pathogenesis of PC, which might be related to the activation of caspases by adiponectin to promote apoptosis and the attenuation of the β-catenin signaling pathway to restrain tumor cell proliferation (30, 31). The tendency of cancer cell growth was inhibited by the treatment of Pan02 mouse PC cells with adiponectin, whereas caspase-3 and caspase-7 activities were significantly augmented in the treated group, thus implying that adiponectin can promote apoptosis in cancer cells (**Table 1****)** (30). Transplantation of Pan02 cells into the pancreas of adiponectin knockout mice resulted in a significant increase in the volume of tumors and a decrease in dUTP nick end-labeling (TUNEL) positive cells, indicating that adiponectin represses the proliferation of PC in mice (30). The downregulation of AdipoR expression was identified by Jiang et al. (31) to eliminate the anti-cancer cell proliferative effect of adiponectin and markedly boosted tumor growth in mice, and further demonstrated that adiponectin reduced cyclin D1 expression by interfering with GSK-3β phosphorylation to prevent its inactivation and degraded β-catenin to inhibit β-catenin accumulation in cells, ultimately blocking PC growth in the G0–G1 phase. This process was coupled with reduced expression of the β-catenin-related transcription factor TCF7L2, providing further evidence that adiponectin can weaken β-catenin signaling and play a critical role in the inhibition of PC growth (31).

### Resistin

First named for its unique ability to induce mice to generate insulin resistance, resistin is otherwise known as adipocyte-specific secretory factor (ADSF) (130) (**Figure 1**). Resistin, which is a peptide hormone encoded by the RSTN gene, belongs to the RELM family along with three types of proteins, RELM-α, RELM-β, and RELM-y (131). Thanks to genetic sequencing, differences between mouse and human resistin, has been demonstrated at the genomic and protein levels. Genomically, the RSTN gene is located on human chromosome 19p13 and mouse chromosome 8A1; at the protein level, human and mouse resistin share only 59% homology, where human resistin is a mature 12.5 kDa molecule consisting of 108 amino acids, while mouse resistin is an 11 kDa polypeptide consisting of 94 amino acids (132). It was initially thought that resistin, like leptin and adiponectin, was mainly secreted by WAT, yet subsequently it emerged that only about one-third of resistin is secreted by WAT in the human body but more often by monocytes and macrophages, which in turn spurs the production of inflammatory factors such as TNF-α, thereby linking adipose tissue to the inflammatory response (133). In this regard, it might be possible that resistin functions as an immunomodulatory factor in the immune response of the TME (134).

Resistin couples four different functional receptors, including adenylylcyclase-associated protein 1 (CAP1), toll-like receptor 4 (TLR4), an isoform of decorin (DCN), and receptor tyrosine kinase-like orphan receptor 1 (ROR1), where DCN and ROR1 bound mainly to murine-derived resistin, while TLR4 and CAP1 were of special interest given their involvement in multiple signaling pathways in humans and their close relationship with the immune response in TME (132, 135). Tarkowski et al. (136) first reported the ability of resistin to interact with TLR4 on the surface of monocytes and epithelial cells as well as to promote the generation of inflammatory factors IL-6, IL-1b, IL-8, and TNF-α, which in turn mediated NF-β and MAPK signaling engaged in the inflammatory response. Following the response of TLR4, it exerts its biological effects mainly through the transcription and secretion of pro-inflammatory cytokines *via* the MyD88 protein-dependent pathway and INF-1 *via* the TRIF-dependent pathway (136). TLR4 has been demonstrated to interact extensively with growth factors such as VEGF, PDGF, and EGF and chemokines such as CXCR7, fulfilling multiple roles in tumor cell apoptosis, metastasis, and microenvironmental immune escape (137). Binding to CAP1, resistin upregulates cAMP concentration in monocytes, which in turn enhances PKA activity as well as promotes transcription of NF-kB-related inflammatory factors (138). Moreover, CAP1 is far from being the only receptor known to bind directly to human resistin, which relates to the CAP1/CAMP/PKA signaling pathway and regulates monocyte function, leading to chronic inflammation (138). The chronic inflammation inspired by TLR4 and CAP1 is involved in the malignancy process, as is the overexpression of TLR4 and CAP1 in the TME, contributing to the progression of cancer. Yamazaki et al. (139) identified overexpression of CAP1 in PC tissues with reduced motility, invasive ability, and lamellar phospholipid formation in cells following knockdown of CAP1, further analyzing that overexpression of CAP1 enhanced nerve infiltration and lymph node metastasis (**Table 1****)**. The study by Yamazaki et al. illustrated the correlation between resistin receptors and PC, and the correlation between resistin itself and PC, as was subsequently demonstrated by Jiang et al. (32) showing that patients with poorly histologically graded PC were often accompanied by resistin(+) with resistin being an independent prognostic factor affecting recurrence-free survival in patients with PDAC.

### Oncostatin

A member of the IL-6 cytokine family, Oncostatin M (OSM), was identified as being able to be produced by AT and was engaged in glucose and lipid metabolism (140) (**Figure 1**). The members of the IL-6 cytokine family all share a common transmembrane subunit, glycoprotein 130 (gp130), in their corresponding receptor complexes, which is why the IL-6 cytokine family is termed the Gp130 cytokine family (141, 142). Oncostatin M, encompassing 252 amino acids and folded into a 4-helix bundle structure, is a special member of the Gp130 family of cytokines in that it features two different receptor complexes compared to others, the gp130/LIFRβ complex and the gp130/OSMRβ complex (141). The gp130/OSMRβ complex represents the main effective receptor of OSM, with the current research in PC focusing on it. In the presence of OSMRβ, OSM could activate specific signaling pathways from STAT5, STAT6, AKT, and PKC-δ, which were unable to be activated by other Gp130 cytokine family members. Upon binding to the gp130/OSMRβ complex, OSM induces transcription of relevant target genes through signaling pathways such as JAK/STAT, MAPK/ERK, and PI3K/AKT with a crucial role in inflammation and hematopoiesis and could act in direct ways as growth inhibitory or growth promoting factors impacting the expansion of tumor cells or indirectly on angiogenesis through the recall of inflammatory cells (141, 143).

As a consequence, different roles are played by OSM in distinct pathological contexts. Comparing the serum expression of 507 cytokines in PC patients and healthy volunteers, five cytokines, including OSM, showed sensitivity ranging from 69 to 77% and specificity of 75% with a highly discriminatory diagnosis of PDAC and exhibited potential as new diagnostic biomarkers of the efficacy of gemcitabine and erlotinib (33). Increased circulating OSM suggests of increased severity of PC and currently understood mechanisms involve the promotion of EMT, regulation of stem cell plasticity, reprogramming of fibroblasts and reinforcement of chemotherapy resistance in PC. Tan et al. (34) were the earliest to report that OSM could induce EMT along with more elongated, fibroblast-like cells, a mechanism that may involve JAK/STAT signaling (**Table 1****)**. The phenotype-driven effects of OSM on mesenchymal and cancer stem cells (CSC) were further investigated by Smigiel et al. (35). Upon exposure to OSM, additional PC cells expressed mesenchymal/CSC properties, and increased tumor metastasis, enhanced tumorigenicity, and resistance to gemcitabine were observed (35). This induction of the MSC/CSC cellular state is maintained by a positive feedback effect resulting from activation of the OSMR/JAK/STAT3 pathway induced by OSMR binding and inhibition of OSM function would prevent PC cells from acquiring and maintaining MSC/CSC properties, thereby preventing cancer cells from participating in a favorable escape mechanism (35).

A tumor-inflammatory microenvironment that promotes tumor growth and metastasis could be established by OSM to affect the prognosis of PC patients, which is achieved by reprogramming inflammatory cancer-associated fibroblasts (iCAF) (36). CAF itself has become the mainstay of PC with an abundance of desmoplastic reactions during tumorigenesis. CAFs with high OSMR expression frequently have a release of inflammatory signals such as CXCL1, CCL2, CCL7, and IL6 (36). Additionally, OSMR was found to contribute to the immunosuppressive microenvironment of PC, with classical monocytes in OSM (−) PC showing attenuated differentiation towards TAM with immunosuppressive properties and increased expression of CD40 and CD86, co-stimulatory molecules expressed by antigen-presenting cells (APCs), contributing to effective activation of T cells (36). LLGL1 augments the sensitivity of PC to gemcitabine and this sensitizing effect has been shown to be related to the induction of an anti-apoptotic effect and CSC by LLGL1 through the regulation of the ERK2/Sp1/OSMR axis (37). Intervening in OSM-OSMR signaling to regulate the iCAF reprogramming process and targeting OSMR to enhance the therapeutic effect of gemcitabine would be novel directions for treating PC (**Table 1****)**.

### Osteopontin

Osteopontin (OPN), a multifunctional adipokine protein, holds the promise of being complementary to CA199 as an early clinical diagnostic marker (38) (**Figure 1**). This is in part, owing to the specific elevated expression of osteopontin in the serum or tissues of PC patients. Chen et al. (39) showed that osteopontin could uniquely discriminate healthy people from cancer. Adding to this, Poruk et al. (40) noticed that serum osteopontin significantly differed in PC patients compared with normal subjects in which elevated osteopontin was positively associated with patient mortality. What is of interest is that in the study by Collins (41), although osteopontin was also detected to be elevated, it was also noted that osteopontin has a protective effect independent of tumor stage. Also, OPN was elevated in PC tissues compared with healthy subjects and the relationship between PC clinicopathological parameters and osteopontin immunoscore was well explained, whereby its expression was indicative of clinicopathological stage level (144) (**Table 1****)**.

Leukocytes and mesenchymal stem cells (MSCs) could be recruited by osteopontin from either peripheral blood or bone marrow (145). Tumor-localized fibroblasts could be reprogrammed by osteopontin to become CAF, as could be M1 anti-tumor macrophages, which are reprogrammed to become TAM (145–147). Osteopontin may also be referred to as secreted phosphoprotein 1 (SPP1) (148). It also belongs to the SIBLING family, which stands for “small integrin binding ligand N-linked glycoproteins” and was initially recognized as being present in bone and dentin, where it fulfills the duties of extracellular matrix (ECM) formation and mineralization (149, 150). Members of this cytokine family enable the regulation of cell signaling by forming a functional complex with multiple integrins and CD44. The binding of integrin receptors during the action of osteopontin depends on the RGD domain (Arg-Gly-Asp) as well as a thrombin-cleaved epitope SVVYGLR (also known as the non-RGD domain), while the cell surface hyaluronan receptor CD44 subtype CD44v (6–15) binds to the calcium binding domain and acts in an RGD-independent manner (151, 152). The OPN–integrin complex activates key downstream molecules such as ERK2, (NF)-κB, IKKβ, and PI3K, while the OPN–CD44 complex is extensively involved in MAPK, FGFR2, PI3K, RAS, Wnt/β-catenin, and Hippo-YAP signaling pathways (151, 153). There may be more potential signaling, transcriptional, and therapeutic sites independent of these signaling pathways in the development process of PC.

Osteopontin promotes metastasis of PC and inhibition or reduction of osteopontin expression effectively inhibits liver metastasis of PC, a function that is exclusive to osteopontin compared to the structurally comparable osteopontins of the same family of SIBLINGs (154–156). The need to effectively halt early metastasis of PC is paramount considering that the high mortality rate of PC is inextricably linked to the early stages of metastasis. Additional causative factors of death from PC that leave clinicians at the end of their wits include the complex hyperplastic connective tissue of PC and, furthermore, increased desmoplasia hyperplasia is a key mechanism by which obesity contributes to the progression of PC, to which pancreatic stellate cells (PSCs) contribute in large part (157). Upon stimulation with TNF-α/β, IL-1/2/10, etc., PSCs can be activated into CAF isoforms like iCAF and myCAF, which participate in the desmoplastic reaction to remodel the mesenchyme of PC (158, 159). Hypoxia in solid malignant tumor cells such as PC is a pivotal ingredient for intensified aggressiveness, metastasis, and angiogenesis. PSCs are capable of being activated at hypoxia with the assistant of ROS and accompany of increased production of osteopontin, which proceeds to bind to integrin avβ3 and activates Akt/Erk signaling and then both regulating EMT processes in PC cells (PCCs) and regulates the expression of CSC-like properties, simultaneously inducing the malignant phenotype in PCCs (42). FOXM1, a proto-oncogene, serves as a downstream effector molecule in the osteopontin regulatory axis (42). In the context of CAF-PCC co-culture, CD44+ cells were significantly increased along with other stemness markers such as ALDH+ and AF+ (43). Moreover, the expression of osteopontin was also increased in the system and a reduction in stemness and tumorsphere formation ability was observed when either osteopontin or CD44 was knocked out, suggesting a role for osteopontin-CD44 in the regulation of stemness (43) (**Table 1****)**. H3K4me3, an essential modification of histone during the process of immune checkpoint inhibitor (ICI) immunotherapy, can act as a promoter to increase osteopontin expression and amplify osteopontin/CD44 axis signaling, thus making osteopontin a novel immune checkpoint that could complement classical PD-1 immune escape and weaken the effect of anti-PD-1 immunotherapy (44). The multiple functions of osteopontin are also manifested by the fact that the expression of osteopontin is elevated in the presence of increased glucose and insulin levels, together with an increased proliferation of pancreatic ductal epithelial cells (45).

## Potential adipokines and pancreatic cancer

Certainly, additional adipokines involved in the malignant progression of PC will be confirmed in coming years. With their diverse cancer-related profiles, three adipokines, kisspeptin, omentin, and chemerin, stand out from the crowd of adipokines, but research in the PC context remains limited. The specific suppressive role of kispeptin demonstrated in other malignancies against tumor migration is similarly seen in PC. Omentin and adiponectin, the previously mentioned unique protective factor, possess parallels in their secretory patterns, opening up the potential for underexplored protective adipokine features of omentin. Chemerin, being a recent star adipokine, might probably possess the type of relationship with tumor immunity and metabolism proven in PC and in other malignancies. Regardless of kispeptin, omentin, or chemerin, in the future, all could present a direction for further reflection on the mechanics of the adipokine role in the context of PC.

### Kisspeptin

As a member of the adipokine family, kisspeptin was originally referred to as metastatic owing to its unique inhibitory ability on tumor migration (**Figure 1**). KISS-1 is a pre-peptide containing 145 amino acids, which are terminally amidated by the serum proprotein convertase Furin to generate numerous bioactive peptides, namely, kisspeptin, including KP-13, KP-54, KP-14, and KP-10 isoforms, among which KP-54 is currently the most studied. On the one hand, these isoforms (KPs) function as regulators of malignant migration, and on the other hand, they are positive regulators of the mammalian reproductive neuroendocrine axis (160). Located on the long arm of chromosome 1, KISS-1 is regulated by TXNIP, CRSP3, and TCF21 and is mainly expressed in tissues such as the placenta, kidney, pancreas, and hypothalamic arcuate nucleus, where it exerts vital roles in the suppression of native tumor metastasis (161) (**Figure 1**). Ongoing studies confirm that the KISS-1 expression pattern significantly overlaps matrix metalloproteinases (MMP), a central mechanism by which the MMP family is involved in the metastatic process of malignant tumors, which explains the tumor metastasis inhibitory role of kisspeptin in an array of tumors (162, 163). KISS-1R, the natural receptor for kisspeptin, also called Gpr54, belongs to a family of seven transmembrane G protein-coupled receptors consisting of α, β, and γ subunits of which are highly distributed in the pancreas, placenta, pituitary gland, and spinal cord and similarly closely associated tumor metastasis (164, 165). Of these, the α subunit contains four initiation signals, including GαS, Gαi/Gαo, Gαq/Gα11, and Gα12/Gα13. Both KISS-1R can bind to Gαq/Gα11 and separately activate the small G protein RhoA, thereby linking to downstream tumor-related signaling pathways like EGFR, CXCL12/CXC4, TNFα, NFκB, PI3K, TGFβ, etc. (163).

While kisspeptin was initially shown to suppress metastasis in melanoma, subsequent studies have found that KISS-1/kisspeptin could boost metastasis in malignancies ranging from liver to breast cancer, suggesting a dual identity for kisspeptin in cancer (166). Kisspeptin still remains to play a cancer metastasis inhibitory role in PC. McNally et al. (167) found that mice implanted with S2VP10L-KISS, PC cells overexpressing KISS-1, had fewer liver and lung metastases from pancreas but showed no significant difference in tumor cell proliferation. Similarly, Masui et al. (46) showed that though exogenous kisspeptin did not inhibit the proliferation of PC cells, it did exhibit a significant influence in reducing the migration ability of PC cells *in vitro*, presumably related to the activation of ERK1 and p38 by kisspeptin **(** **Table 1****)**. Wang et al. (47) observed that PC cells PANC-1 with high KISS-1R expression exhibited similar tumor migration inhibition to BxPC-3 cells with low KISS-1R expression after transfection with KISS-1, implicating that KiSS-1 overexpression-mediated invasion inhibition was not dependent on the receptor expression level. Multiple current studies confirmed that although KISS-1 is less expressed in PC tissues than in normal, PC patients with high KISS-1R expression in their tumors tend to have higher survival rates and better prognosis. Contrary to the diminished expression of kisspeptin in tumor tissue, the current clinical studies revealed a significant increase in serum kisspeptin expression (48). Likewise, Katagiri et al. (49) came to this conclusion and explained that serum kisspeptin may be secreted by normal body tissues as a “self-defense mechanism” against cancer progression. As a result, serum kisspeptin levels can be of translational medical value as a non-invasive prognostic indicator for patients with PC. Additionally, the exploitation of antibiotic agents targeting the KISS-1/Kispeptin/KISS-1R axis may be a viable strategy for adjuvant PC treatment. Examples include alpha-bisabolol, an effective inhibitor of tumor aggressiveness in PC, whose cancer-suppressive effects were previously proved relevant to the activation of KISS-1R, giving rise to new ideas for the clinical treatment of PC (50).

### Omentin

Omentin is an accepted hydrophilic adipokine with two highly homologous isoforms (20), omentin-1 and omentin-2, which share 83% amino acid homology (168) (**Figure 1**). Omentin-1 is the predominant circulating form of omentin, originally identified in small intestinal Paneth cells and endothelial cells as intelectin-1, which is abundant in human plasma and referred to as the galactofuranose binding lectin, intestinal lactoferrin receptor, and endothelial lectin (169). As demonstrated by experimental evidence, omentin-1 preserves body metabolism as well as enhances insulin sensitivity and exerts anti-inflammatory, anti-atherogenic, and tumor growth-regulating effects through AMPK, AKT, NF-κB, MAPK, ERK, JNK, and p38 signaling. Remarkably, the level of omentin-1 in plasma was positively correlated with adiponectin, which has been shown to inhibit the growth of many malignancies as an adipokine with reduced levels in obese people, suggesting that there might be a similar form of regulation between omentin-1 and adiponectin, all of which provide directions for further dissection of the mechanisms underlying the regulation of PC growth by omentin (51). For instance, omentin-1 inhibits the proliferation and promotes the apoptosis of colon CSCs through the PI3K/Akt pathway, which is apparent depending on the duration and concentration of exposure (170). There are no binding receptors for omentin identified, and some scholars speculate that it might be a non-protein component of the cell surface, such as a carbohydrate or glycolipid (168). Contemporary research on omentin and PC has been limited to clinical studies. Karabulut et al. (52) analyzed serum samples from 33 PC patients and found PC patients had significantly higher baseline levels of serum omentin compared to controls (p <0.001) and that an identical result was seen in patients with larger pathological tumor volumes. Kiczmer et al. (53) also detected a significant increase in omentin-1 levels in patients with PDAC. Arjmand et al. (54) stated that omentin levels were markedly associated with the risk of PC and that increased serum levels of omentin-1 may result from the response of body to the tumor, although the exact mechanism by which omentin regulates PC is still unclear (**Table 1****)**.

### Chemerin

The function of chemerin to activate signaling within tumor cells, enhance natural and acquired immune defense against tumors and promote endothelial angiogenesis has made deciphering the relationship between chemerin and various malignancies a hot topic of research in oncology (171) (**Figure 1**). Chemerin, also known as tazarotene inducible gene 2 (TIG2) or retinoic acid receptor response protein 2 (RARRES2), is produced by C-terminal processing of pro-chemokines secreted by the liver and WAT (172). To a large extent, human chemerin isoforms include chemerin 156, chemerin 157, and chemerin 158, of which vhemerin 157 represents the most dynamic variant (173).

The dual role of chemerin in cancer development is that high serum chemerin levels may indicate increased survival in patients with tumors such as breast cancer, ovarian cancer, hepatocellular carcinoma (HCC), and adrenocortical carcinoma (ACC), while high chemerin expression suggests poor prognosis in patients with gastric cancer and non-small cell lung cancer (NSCLC) (171). For PC, Kiczmer et al. (53) evaluated serum chemerin levels in 27 patients with PC, 10 patients with CP, and 36 control volunteers and found that chemerin levels were significantly elevated in patients with PC (**Table 1****)**. Unfortunately, the current study did not disclose the expression of chemerin in PC tissues and did not further investigate the relationship between chemerin expression and the prognosis of PC patients. The mechanisms by which chemerin exert their cancer suppressing or promotive effects differ, with recruitment of immune cells causing them to inhibit tumor growth, and induction of neovascularization allowing chemerin to facilitate tumor growth (174). There exist three known receptors for chemerin, namely chemokine-like receptor 1 (CMKLR1), G protein-coupled receptor 1 (GPR1), and C-C chemokine receptor-like 2 (CCRL2) (175) (**Figure 1**). The former two are capable of transmitting signal transduction and have been respectively named Chemerin_1_ and Chemerin_2_ (172). Additionally, CMKLR1 has also been referred to in other literature as ChemR23. CCRL2 is thought to be a non-signaling receptor with a high affinity towards chemerin and, while unable to transmit signals *via* any known signaling pathway, CCRL2 is able to bind and deliver chemerin to establish concentration gradients and thus get involved in various functions (172, 176). Recently, Delbany et al. (177) reported the role of chemerin as a negative regulator of tumorigenesis in a skin cancer model, where the rate of papilloma development was significantly elevated upon loss of CCRL2 and this effect was abolished in the absence of CMKLR1, suggesting that the biological function of CCRL2 is closely dependent on CMKLR1. Also in this study, tumor cells overexpressing CCRL2 were found to aggregate chemotactic proteins, condensing chemerin on the cell surface, thus promoting activation of CMKLR1-expressing cells (177). To further demonstrate that these anti-tumor effects of CCRL2 are mediated by local concentrations of chemerin. In contrast, unlike other tumors, chemerin is able to inhibit the generation of skin cancer vessels, thereby inducing cell death and delayed proliferation (177). Chemerin is involved in the regulation of the TME through recognition of cell surface CMKLR1 displayed by macrophages, NK cells, endothelial cells, myeloid and plasmacytoid dendritic cells, smooth muscle cells, and adipocytes that express CMKLR1, a subpopulation of cells present in the TME (178, 179). Moreover, chemerin has recently been linked to tumor lipid metabolism and ferroptosis. Tan et al. (180) found that deregulation of chemerin expression inhibited tumor growth and significantly reduced intracellular lipid deposition in CcRCC cells. Further analysis revealed that lipid metabolism was reprogrammed in cancer cells after inhibition of chemerin expression and enhanced lipid oxidation resulted in increased susceptibility to ferroptosis in CcRCC cells (180). Concurrently, it was observed that lipid coenzyme Q and mitochondrial complex IV were both reduced following chemerin inhibition, leading to lipid reactive oxygen species production and further promoting ferroptosis (180). Taking these mechanisms together into account could provide a breakthrough idea to explore the rationale for chemerin in PC.

## Conclusion

Concurrently, obesity serves as a recognized risk factor for cancer. Despite the increasing knowledge of obesity-related malignancies, the specific mechanisms underlying the interconnections seem to be complex. Deciphering the mechanisms involved and finding the value of translational medicine is an increasingly critical topic in oncology. Adipokines are of increasing interest as key mediators influencing the pathophysiology ascribed to cancer.

Hence, throughout this review, we highlight the important role of AT and adipokines in the pathology of PC (**Table 1****)**. As demonstrated in **Table 1**, with the exception of adiponectin, all the representative adipokines listed in this review at the present time have the ability to promote malignant progression of PC. Although adiponectin has shown divergent results in clinical studies, *in vivo* or *in vitro* studies have all confirmed its antagonistic impact on PC. Such a distinct oncogenic effect of adiponectin is interesting, yet this mechanism is currently not well documented in PC. Furthermore, through the review, we found that although all being members of the adipokine family, the various adipokines mentioned above induce different pathophysiological processes in PC through a diverse range of mechanisms, ultimately altering the results of tumor as well as the prognosis of tumor patients. Hence, we pose the question of whether classical adipokines including resistin and adiponectin interfere equally profoundly with the course of PC by regulating TME, tumor metabolic reprogramming, and other pathways, which needs to be further determined by future studies.

We also found that many other adipokines that show regulatory effects in other GI cancers, like visfatin and apelin, have a gap in research in the context of PC and need to be expanded by further studies, both in clinical studies and in an in-depth exploration of the mechanisms involved. As **Table 1** collated, several adipokines are mentioned in the review, such as chemerin and omentin. There are still lots of gray areas that exist in the mechanism of their action in PC, and it may be possible to look at the mechanisms listed above, in other malignancies, for ideas as well as directions. This is notable since we conjecture that adipokines act as a bridge between obesity and cancer, while often acting as an endocrine hormone involved in the metabolic regulation of the body. It is likely that multiple distinct adipokines cascade with each other to form a feedback mechanism and thus a web of interactions. Identifying this complex and variable matrix of associations is likely to yield specific endocrine therapeutic avenues that are critical to improving the clinical malignancy treatment landscape.

Furthermore, the translational medical value of adipokines includes being a diagnostic marker of specificity. We have seen this potential in osteopontin, leptin, and others. With further exploration of multiple adipokine combination diagnostic modalities in the future, this role may be maximized. Additionally, such values can be reflected in accurate screening, specifically for early-stage cancers in the obese population, which is likely to significantly improve disease prognosis in them. In addition to the role of diagnostic markers, bringing together multiple adipokines for use as a grading scale and tailoring different treatment protocols for obese patients at different stages may significantly improve their survival rates. Simultaneously, there is mounting support for the idea that the adipocyte-rich microenvironment plays an instrumental role in the development of various tumors. Adipokines can act locally within AT and systemically, equally regulating the TME, which means future exploration of such mechanisms is of equal value. And, we conjecture that, as an endocrine factor, adipokines are most likely also engaged in tumor metabolic reprogramming, as they regulate metabolism systemically, but unfortunately, although we currently see this role in newly identified adipokines, this is currently limited and further *in vivo* and *in vitro* experiments are needed to validate this. In summary, adipokines have great potential to become reliable targets for neoadjuvant tumor therapy, and the prospect of such applications is compelling.

## Author Contributions

QW prepared the figure and drafted this manuscript. QW, HW, YD, MW, and MX conceived the idea and analyzed the literature. MX edited and revised manuscript. All authors contributed to the article and approved the submitted version. All authors listed have made a substantial, direct, and intellectual contribution to the work and approved it for publication.

## Funding

This study was supported by the National Natural Science Foundation of China (No. 82072754), the Jiangsu Provincial Key Research and Development Program (No. BE2018689), the Natural Science Foundation of Jiangsu Province (No. M2020011), and the Zhenjiang Key Research and Development Program (No. SH2018033).

## Conflict of Interest

The authors declare that the research was conducted in the absence of any commercial or financial relationships that could be construed as a potential conflict of interest.

## Publisher’s Note

All claims expressed in this article are solely those of the authors and do not necessarily represent those of their affiliated organizations, or those of the publisher, the editors and the reviewers. Any product that may be evaluated in this article, or claim that may be made by its manufacturer, is not guaranteed or endorsed by the publisher.
